# The role of dendritic cells for therapy of B-cell lymphoma with immune checkpoint inhibitors

**DOI:** 10.1007/s00262-020-02767-6

**Published:** 2020-11-03

**Authors:** Anne Scheuerpflug, Fatima Ahmetlić, Vera Bauer, Tanja Riedel, Martin Röcken, Ralph Mocikat

**Affiliations:** 1 Helmholtz-Zentrum München, Eigenständige Forschungseinheit Translationale Molekulare Immunologie, Munich, Germany; 2 Helmholtz-Zentrum München, Institut Für Molekulare Immunologie, Munich, Germany; 3 Klinik Für Dermatologie, Eberhard-Karls-Universität Tübingen, Tübingen, Germany

**Keywords:** Immune checkpoint blocking, Lymphoma, Tumor-infiltrating dendritic cells, Interferon-γ, λ-MYC mouse

## Abstract

Immune checkpoint blocking (ICB) is a promising new tool of cancer treatment. Yet, the underlying therapeutic mechanisms are not fully understood. Here we investigated the role of dendritic cells (DCs) for the therapeutic effect of ICB in a λ-MYC-transgenic mouse model of endogenously arising B-cell lymphoma. The growth of these tumors can be effectively delayed by antibodies against CTLA-4 and PD-1. Tumor-infiltrating DCs from mice having received therapy showed an upregulation of costimulatory molecules as well as an augmented IL-12/IL-10 ratio as compared to untreated controls. Both alterations seemed to be induced by interferon-γ (IFN-γ), which is upregulated in T cells and natural killer cells upon ICB. Furthermore, the enhanced IL-12/IL-10 ratio, which favors Th1-prone antitumor T-cell responses, was a consequence of direct interaction of ICB antibodies with DCs. Importantly, the capability of tumor-infiltrating DCs of stimulating peptide-specific or allogeneic T-cell responses in vitro was improved when DCs were derived from ICB-treated mice. The data indicate that ICB therapy is not only effective by directly activating T cells, but also by triggering a complex network, in which DCs play a pivotal role at the interface between innate and adaptive antitumor responses.

**Electronic supplementary material:**

The online version of this article (10.1007/s00262-020-02767-6) contains supplementary material, which is available to authorized users.

## Introduction

In the past years, immune checkpoint blockade (ICB) has substantially advanced the field of cancer immunotherapy in the clinics. Approaches have been conceived to block counter-regulatory molecules like cytotoxic T-lymphocyte-associated protein-4 (CTLA-4) or programmed death-1 (PD-1), which are often associated with tumor-infiltrating T cells (TILs) whose functions are silenced or exhausted in the tumor microenvironment [1–6]. Although monoclonal antibodies (mAbs) targeting CTLA-4 or PD-1, such as Ipilimumab or Nivolumab and Pembrolizumab, have already been successfully established as new tools for treating several types of cancer including lymphoma (reviews in [7–10]), the underlying tumor-suppressive mechanisms are not yet completely understood.

While the rationale of ICB originally aimed at re-activating TILs against malignant cells by directly disrupting the interaction between the counter-regulatory receptors on T cells and their ligands, other pathways and cell populations are also involved in mediating the therapeutic effect. Thus, in a λ-MYC transgenic mouse model of endogenously arising B-cell lymphoma [11], ICB-induced long-term tumor suppression was not only due to the activation of T cells, but also to natural killer (NK) cells [12]. Interferon-γ (IFN-γ) and tumor necrosis factor (TNF) were strictly required for tumor control in this model [13]. These cytokines are capable of activating the p16^Ink4a^-Rb and the p53-p21 signaling pathways [13, 14], thereby causing cell cycle arrest of cancer cells [14, 15]. Hence, the effect of ICB was additionally mediated by cytokine-induced senescence of malignant cells [12, 13]. IFN-γ, which was required for this pathway, was not only provided by T cells upon ICB, but also by NK cells, which turned out to be critical for the therapeutic efficacy in the lymphoma model. The NK cells were directly stimulated by the anti-CTLA-4/anti-PD-1 mAbs to produce IFN-γ, thereby contributing to senescence induction in tumor cells [12].

Generally, NK-cell activation can initiate a cascade that bridges the innate and the adaptive immune response. Activated NK cells can lyse tumor cells [16–18], which leads to engulfment of antigens by dendritic cells (DCs) and presentation of immunogenic peptides to T cells in a major histocompatibility complex- (MHC-) restricted manner. In addition, the cytokine IFN-γ, which is upregulated in activated NK cells, stimulates DCs to express IL-12, which eventually fosters a long-lasting T-cell-dependent antitumor memory response [19, 20]. Although IFN-γ expression by NK cells is severely impaired in untreated endogenous lymphoma [21], the NK/DC/T-cell axis can be triggered even in this setting [16] and might also become effective after ICB-induced IFN-γ upregulation.

The expression pattern of the instructive cytokines IL-12 and IL-10 in DCs is decisive for directing immune responses towards a Th1 or Th2 type. Tumor-infiltrating DCs (TIDCs) frequently undergo a shift from IL-12 to IL-10 expression [22–24] (review in [25]). This bias may contribute to immune escape of tumors because T-cell-mediated tumor rejection requires a Th1-driven response involving IFN-γ and IL-12 rather than a Th2-prone milieu, which is promoted by IL-10 [14, 15, 26, 27].

Having previously shown that multiple mechanisms, which also involve NK cells, are elicited by ICB therapy, we now asked the question whether DCs contribute to the therapeutic potential of anti-CTLA-4 and anti-PD-1 mAbs in the λ-MYC lymphoma model. We show that DC functions are favorably affected by ICB, an effect that is at least partially dependent on IFN-γ and may endorse adaptive antitumor immune responses.

## Materials and methods

### Animal experiments

C57BL/6 wildtype (WT) as well as λ-MYC mice were bred in our own animal facility. BALB/c mice were purchased from Charles River Laboratories, and OT-II mice were kindly provided by Prof. Dr. Brocker, Institute of Immunology, München. Breeding of mice and all experiments were only done after approval by the responsible authority.

For ICB therapy, λ-MYC Mice were injected i.p. with 100 µg each of anti-PD-1 (J43; BioXCell, West Lebanon, U.S.A.) and anti-CTLA-4 mAbs (UC10-4B9; BioLegend, San Diego, U.S.A.) starting at an age of 60 days. Treatment was repeated three times every 10 days. To neutralize IFN-γ during ICB therapy, mice received 500 µg, 300 µg, 150 µg and again 150 µg of the anti-IFN-γ mAb XMG-1.2 (Core facility mAb, Helmholtz-Zentrum München) i.p. 6–12 h before each ICB mAb injection.

Depletion of NK cells was performed by i.p. application of 50 μg polyclonal anti-Asialo-GM1 (Thermo Fisher Scientific, Waltham, U.S.A.) in weekly to biweekly intervals. Effective NK-cell depletion was verified by examining splenocytes for NK1.1 expression.

### Fluorescence-activated cell sorting (FACS)

After homogenizing spleens, cells were treated with erythrocyte lysis buffer, meshed through a 35-μm cell strainer, washed with PBS, and analyzed by FACS.

DCs were phenotyped by using fluorochrome-labeled mAbs directed against CD11c (N418; BioLegend), CD80 (16-10A1; BD Pharmingen, Heidelberg, Germany), CD86 (PO3; Thermo Fisher Scientific), PD-1 (J43, Thermo Fisher Scientific), CTLA-4 (UC10-4B9; BioLegend), CD83 (Michel-19; BioLegend), CD40 (3/23; BioLegend), MHCII (2G9; BD Pharmingen) and PD-L1 (MIH6; AbD Serotec, Puchheim, Germany). Dead cells were excluded using the LIVE/DEAD™ Fixable Blue Dead Cell Stain Kit (Thermo Fisher Scientific). Following mAb labeling for 30 min at 4 °C and final washing, cells were analyzed on an LSR II flow cytometer.

To measure intracellular cytokines, cells were stimulated with PMA and ionomycin (1 μg/ml each; Sigma-Aldrich, Taufkirchen, Germany) for 4 h in the presence of 3 μg/ml Brefeldin A (Thermo Fisher Scientific), labeled for CD11c, subjected to fixation and permeabilization and subsequently stained with mAbs against IL-10 (JES5-16E3; BD Pharmingen) and IL-12 (C15.6; BD Pharmingen). IFN-γ in T cells was measured by using fluorochrome-labeled anti-IFN-γ (XMG-1.2, BioLegend) and counterstaining with anti-CD4 (RM4-5, eBioscience, Frankfurt, Germany).

### Functional assays in vitro

Immune cells were isolated from tumor-infiltrated as well as WT spleens. DCs and T cells were purified by immunomagnetic separation using anti-CD11c microbeads (Miltenyi Biotec, Bergisch-Gladbach, Germany) and the CD4^+^ T Cell Isolation Kit (Stemcell Technologies, Vancouver, Canada), respectively. Purity was > 98%.

5 × 10^5^ DCs derived from λ-MYC spleens were incubated in 200 μl RPMI 1640 medium supplemented with 10% FCS in the presence of 0.1 µg/ml each of anti-PD-1 and anti-CTLA-4 mAbs for 72 h in 96-well plates. Then, IL-12 and IL-10 expression was determined by FACS analyses as described above.

For peptide-specific stimulation, 5 × 10^4^ DCs from untreated or ICB-treated λ-MYC or from WT mice were incubated with MHC II-restricted OVA_323-339_ peptides (GenWay Biotech, San Diego, U.S.A.) at a concentration of 1 μg/ml for 3 h. Then, peptide-pulsed DCs were coincubated with 10^5^ CD4^+^ T cells derived from OT-II mice. For allostimulation, 10^4^ DCs freshly isolated from untreated or ICB-treated λ-MYC or from WT mice were cocultured with 2 × 10^5^ T cells from BALB/c mice.

All T-cell stimulation experiments were done in 200 μl RPMI 1640 medium supplemented with 10% FCS in 96-well plates. Supernatants were taken after 3 and 5 days and analyzed for IFN-γ concentrations by enzyme-linked immunosorbent assay using the Mouse IFN-γ ELISA Ready-SET-Go kit (eBioscience).

### Statistics

Differences between two independent groups were assessed using the unpaired Student’s *t* test or Mann–Whitney test. All results were expressed as means ± SEM. Data analysis was performed with Prism 5.0 software (GraphPad).

## Results

### Increased expression of costimulatory molecules on TIDCs after ICB

λ-MYC mice, which are of C57BL/6 origin, constitutively express a transgenic *c-MYC* oncogene in a B-cell-specific manner, which leads to the development of endogenous B-cell lymphomas in 100% of mice [11]. Tumors grow in spleen and lymph nodes and lead to death between about day 70 and day 140 after birth. As shown earlier, DCs in the tumor microenvironment show phenotypic and functional alterations such as a reduced IL-12/IL-10 ratio and an impaired capability of stimulating T cells, although the expression of costimulatory molecules was increased [24]. Furthermore, the fraction of CD11c^low^ DCs, which are considered as a T-cell-inhibiting DC subset [28], was increased in comparison to the CD11c^high^ population.

We furthermore showed that combined treatment with anti-PD-1 and anti-CTLA-4 mAbs significantly delays tumor development and even gives rise to long-time survivors, while therapy with single mAbs remains uneffective [13]. The frequencies of T lymphocytes, which are greatly diminished in the tumors, partly recover by ICB therapy [12]. While in diseased animals, the organ architecture is destroyed, lymphoid organs from ICB-treated mice, which are still healthy, show a normal distribution of T and B cells [13].

**Fig. 1:**
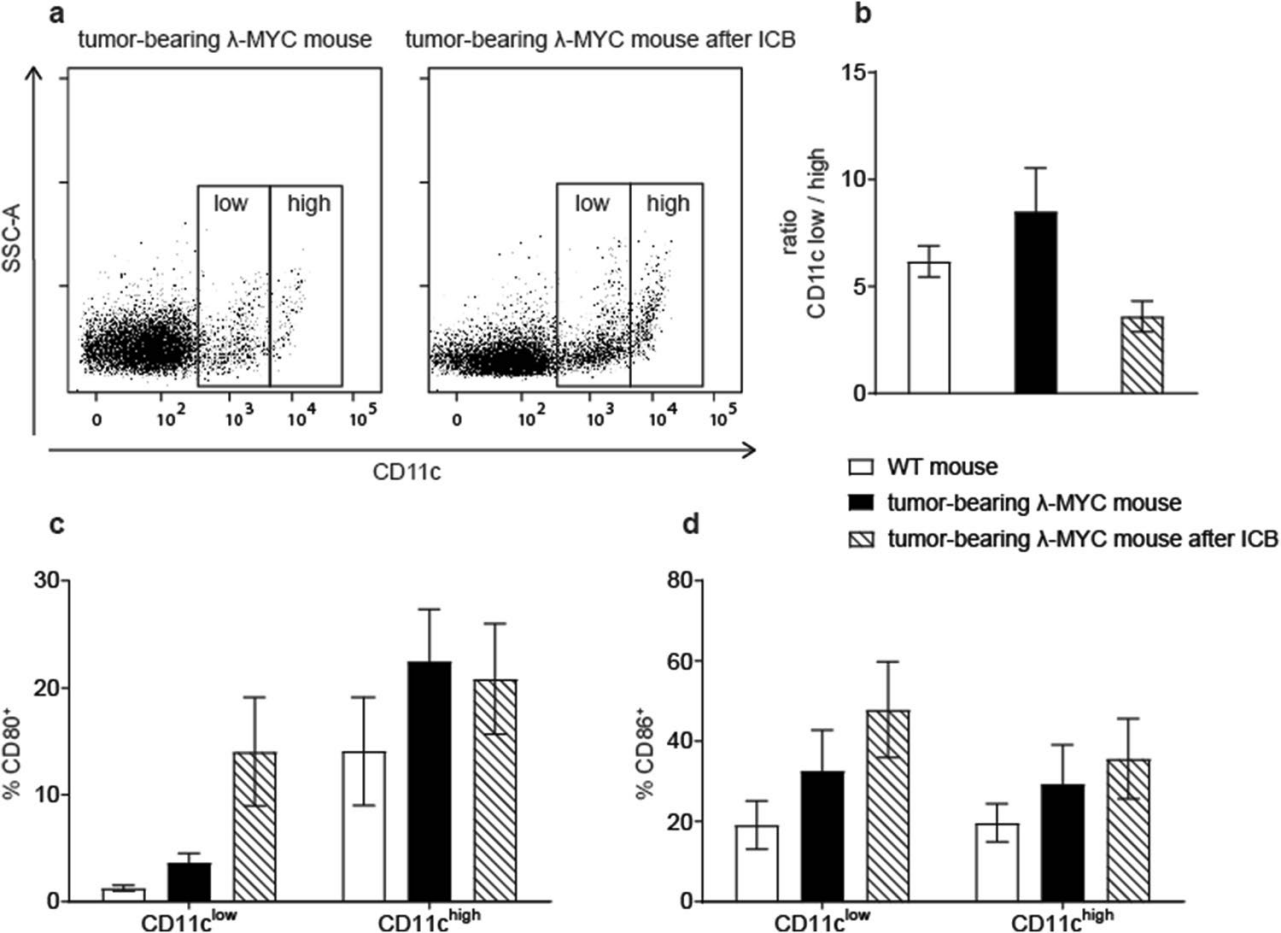
Characterization of surface markers expressed on spleen DCs from tumor-bearing λ-MYC mice that did or did not receive ICB treatment. **a** Distribution of CD11c^low^ and CD11c^high^ DCs. In the example shown, the ratio CD11c^low^/CD11c^high^ is 5.12 (untreated) and 2.87 (treated). **b** Compilation of CD11c^low^/CD11c^high^ ratios from untreated (*n* = 24) and treated mice (*n* = 7). WT mice are included for comparison. The difference between the treated and the untreated group is significant with *P* < 0.05. **c**, **d** Percentages of CD80^+^ and CD86^+^ cells in the CD11c^low^ and CD11c^high^ fractions. 4 and 9 animals were analyzed in the ICB and in the control group, respectively. In the CD11c^low^ population, the differences are significant with *P* < 0.05

The therapeutic effect was associated with increased IFN-γ release by T cells as well as by NK cells [12]. To elucidate the impact of ICB on DCs, we phenotypically characterized spleen-derived TIDCs from mice that received ICB therapy and therefore showed delayed tumor growth. The comparison with untreated animals, whose tumor burdens were identical but only appeared at earlier time points than in the treated group, revealed that ICB significantly reduced the ratio of CD11c^low^ to CD11c^high^ cells (Fig. 1a, b). As the costimulatory molecules CD80 and CD86 expressed on DCs, which are most important for activating specific T cells and preventing T-cell anergization, can be upregulated by IFN-γ [24], we particularly analyzed these molecules on TIDCs. It turned out that ICB further increased the expression of CD80 and CD86, at least in the CD11c^low^ subset (Fig. 1c, d). With regard to other maturation-associated markers, ICB showed variable effects. While slight increases were detected for CD40 and MHC class II, CD83 was markedly reduced after ICB, a finding that cannot be explained for the time being (Supplementary Fig. 1). Of note, PD-L1, which interacts with PD-1 and thereby impairs T-cell functions, was significantly reduced on CD11c^low^ TIDCs from ICB-treated mice.

### ICB affects the pattern of instructive cytokines expressed by TIDCs via IFN-γ

**Fig. 2:**
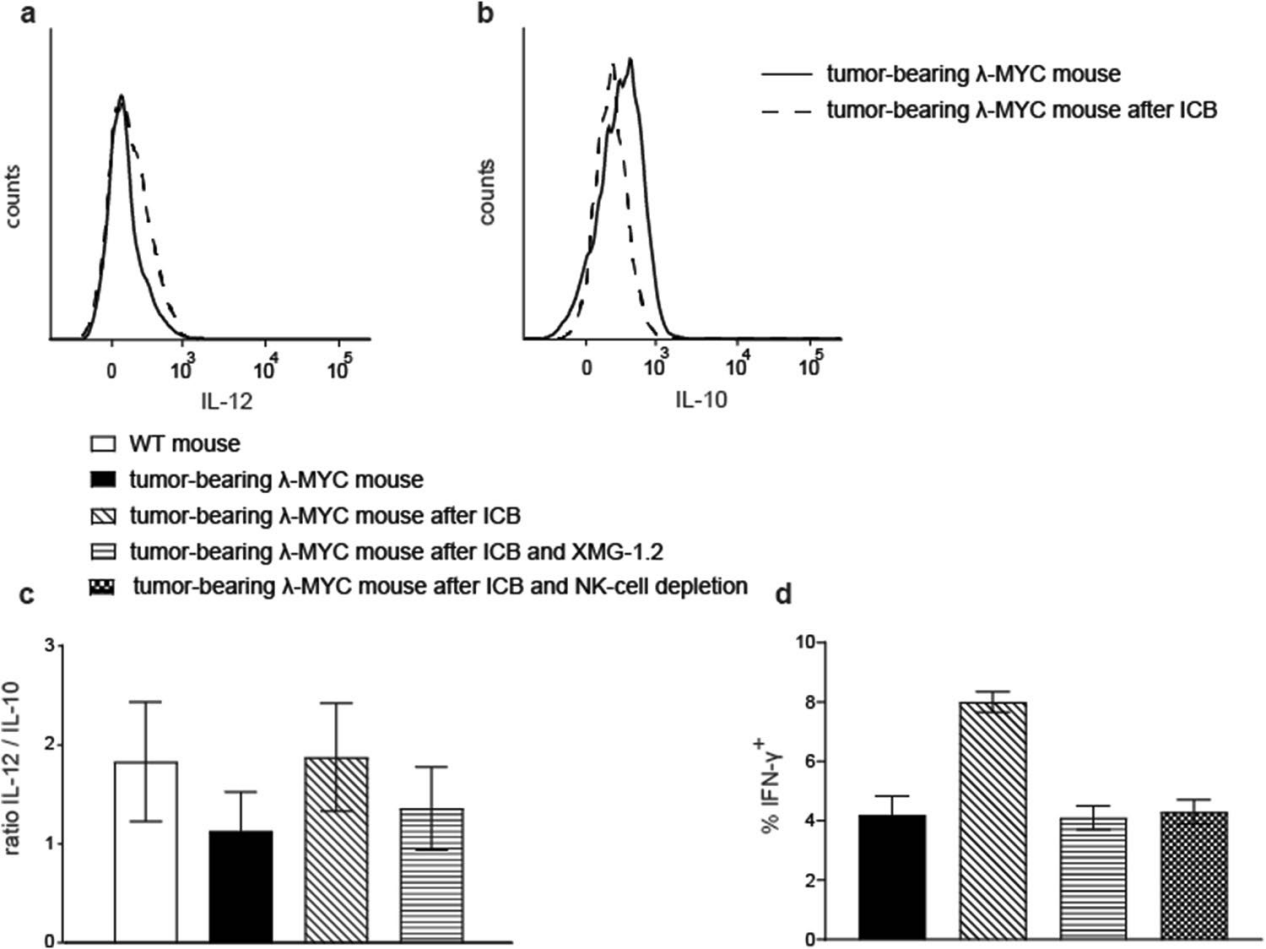
Impact of ICB therapy on expression patterns of instructive cytokines in TIDCs. **a**, **b** Typical result of intracellular staining of IL-12 and IL-10 in CD11c^+^ spleen cells. Similar patterns were detected in CD11c^low^ and CD11c^high^ cells. **c** Compilation of IL-12/IL-10 ratios in TIDCs from animals that received ICB therapy or were left untreated. In some animals, ICB was combined with neutralization of IFN-γ using the mAb XMG-1.2. Up to 14 mice were used per group. **d** Percentages of IFN-γ^+^ cells in the CD4^+^ T-cell population from spleens of the indicated mouse groups. 4–5 mice were included in each group. The difference between the ICB-treated group and all other settings is significant with *P* < 0.01. In the CD8^+^ subset, the effect of ICB in terms of IFN-γ induction was far less pronounced [12]

The expression profile of instructive cytokines in TIDCs from untreated tumor mice displayed a reduced IL-12/IL-10 quotient, which predicts a compromised Th1/Tc1 polarization of T-cell responses [24]. To answer the question whether ICB affects this imbalance of DC-derived instructive cytokines, we analyzed intracellular cytokine levels in TIDCs from spleens of ICB-treated λ-MYC animals. After ICB, individual mice showed variable results with increased IL-12 and reduced IL-10 expression (Fig. 2a, b), which resulted in an increased IL-12/IL-10 ratio, even though this was not statistically significant (Fig. 2c).

The decreased IL-12/IL-10 ratio in DCs from untreated tumor-bearing mice is likely a consequence of IFN-γ suppression, which occurs in the microenvironment of growing λ-MYC lymphomas [21]. Since enhanced amounts of IFN-γ were provided by NK [12] and T cells (Fig. 2d) after delivery of anti-PD-1 and anti-CTLA-4 mAbs, we speculated that the higher IL-12/IL-10 ratio following ICB therapy is induced by this cytokine. Therefore, we analyzed λ-MYC mice that were treated with ICB under mAb-dependent neutralization of IFN-γ. After blocking of IFN-γ, the ICB-induced increase of the IL-12/IL-10 ratio in TIDCs was indeed attenuated (Fig. 2c). This suggests that IFN-γ plays a role for transmitting ICB-dependent effects to DCs. Depleting IFN-γ during ICB therapy also resulted in an impaired T-cell-intrinsic capability of producing IFN-γ (Fig. 2d). As the same result was obtained by depleting NK cells (Fig. 2d), the latter cell population seems to be necessary for providing IFN-γ, which, in turn, supports the T-cell response. IFN-γ may exert this effect directly, but presumably also through alteration of instructive cytokines derived from DCs.

### Direct interaction of ICB mAbs with DCs

**Fig. 3:**
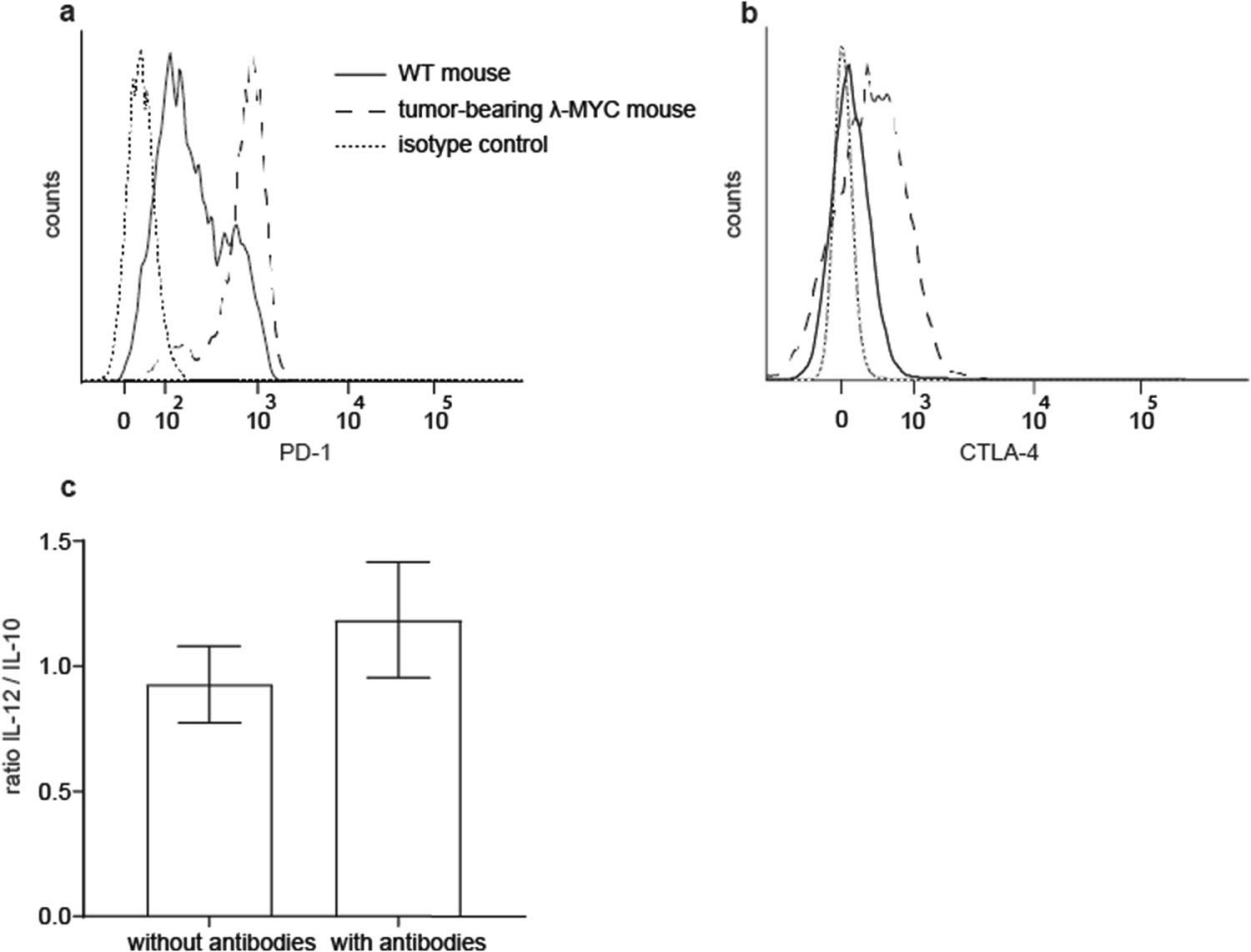
Direct interaction of anti-PD-1 and anti-CTLA-4 mAbs with DCs. **a**, **b** Exemplary results showing higher levels of PD-1 and CTLA-4 expressed on λ-MYC TIDCs than on DCs from WT mice. **c** IL-12/IL-10 ratios in purified CD11c^+^ cells with or without incubation with ICB mAbs for 72 h in vitro (*n* = 5). Identical results were obtained in CD11c^low^ and CD11c^high^ cells

These results did not preclude that direct binding of ICB mAbs to the DC surface may also occur. Therefore, we tested first the expression of immune checkpoint molecules by TIDCs. In comparison to WT DCs, TIDCs from λ-MYC animals exhibited strong expression of PD-1 and CTLA-4 (Fig. 3a, b). This finding prompted us to determine the cytokine profile of purified CD11c^+^ DCs after incubation with anti-PD1 and anti-CTLA-4 mAbs in vitro. After mAb treatment, the IL-12/IL-10 quotient was indeed enhanced (Fig. 3c). As enrichment of DCs via CD11c also yields low numbers of T and NK cells expressing CD11c (not shown), IFN-γ secreted by the latter cells may be responsible for this effect, but it cannot be ruled out that ICB mAbs may have impact on TIDC-derived instructive cytokines by direct interaction with DC surface molecules.

### Functional restoration of TIDCs by ICB therapy

**Fig. 4:**
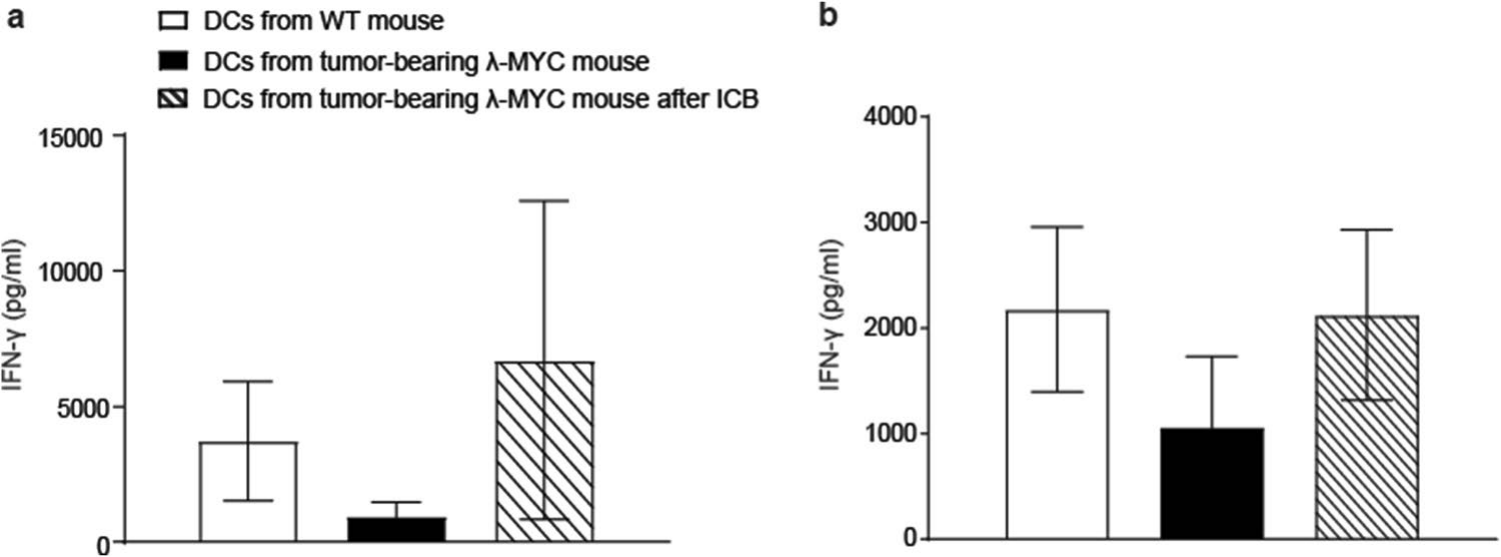
In-vitro stimulation of CD4^+^ T cells by DCs from WT mice or from tumor-bearing MYC animals without or after ICB therapy. **a** OT-II-specific T cells were stimulated with OVA peptide-loaded DCs (*n* = 3). **b** CD4^+^ T-cell response after allogeneic stimulation (*n* = 6). Supernatants were analyzed for IFN-γ concentrations after 5 days. The WT and the MYC groups without therapy are different from the ICB groups with *P* < 0.05

Since the T-cell-stimulatory capacity of λ-MYC tumor-derived TIDCs was compromised [24], we asked the question whether the tumor-dependent functional impairment of DCs can be attenuated by ICB. Therefore, we examined the function of DCs from ICB-treated mice in terms of stimulating naïve T cells in vitro. For testing peptide-specific reactivity, we incubated syngeneic OT-II CD4^+^ T cells, which express OVA-specific T-cell receptors, with TIDCs that were pulsed with appropriate MHC class II-restricted OVA peptides. Whereas IFN-γ secretion by T cells was impaired when stimulated with TIDCs from untreated λ-MYC mice, IFN-γ production by CD4^+^ T cells was restored when DCs were derived from donors that had been subjected to ICB therapy before (Fig. 4a). Similar results were obtained when normal CD4^+^ T cells were stimulated in an allogeneic setting using BALB/c mice as donors of responder cells (Fig. 4b).

## Discussion

**Fig. 5:**
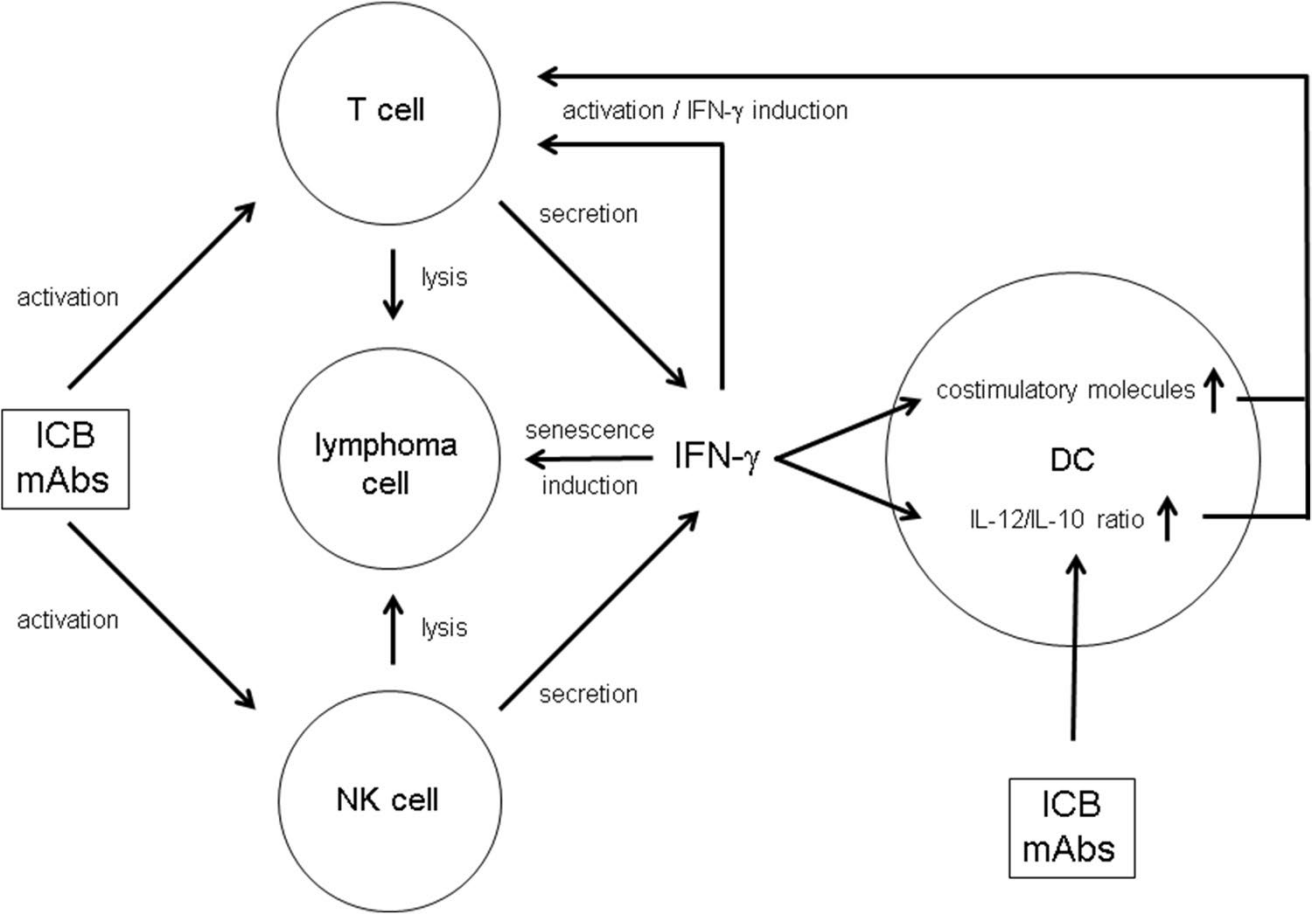
Putative role of DCs and IFN-γ for mediating the effect of ICB mAbs in the therapy of lymphoma. For details see text

The mechanisms underlying ICB-mediated tumor suppression are complex (Fig. 5). In the λ-MYC lymphoma model, we showed that IFN-γ is strictly required for successful therapy [12, 13]. This cytokine not only fosters tumor-protective Th1/Tc1 responses but also triggers several non-immunologic pathways leading to tumor growth inhibition [13, 14, 29] including intrinsic senescence induction in tumor cells [12–14]. Furthermore, NK cells activated by ICB were necessary for prolonging the survival of λ-MYC mice [12]. NK cells may be a critical source of IFN-γ that was needed for the therapeutic effect. The data presented here suggest that DCs are also involved in ICB therapy and that activation of an NK cell/DC axis may additionally endorse tumor-directed T-cell responses (Fig. 5).

The maturation status of TIDCs, hence the expression of costimulatory molecules like CD80 and CD86, seems to be determined by the tumor entity. Thus, in some endogenous mouse tumor models, DCs displayed a downregulation of costimulatory molecules [23, 30], whereas a mature phenotype was observed in other neoplasias including the λ-MYC lymphoma [24, 31]. It has been shown that soluble IFN-γ or IFN-γ-producing NK cells are capable of increasing the CD80 and CD86 expression on DCs [24]. Therefore, it was not surprising that ICB-dependent IFN-γ induction in T cells and in NK cells resulted in an even higher density of CD80 and CD86 on TIDCs (Fig. 1c, d, Fig. 5). However, it is an open question whether this additional upregulation is relevant for the success of ICB therapy, because, in untreated tumor-bearing λ-MYC mice, costimulatory molecules on DCs were also elevated in comparison to normal mice and an anergization of T cells owing to a lack of costimuli is, therefore, unlikely to account for immune escape [24].

Instead, a beneficial effect might be mediated by an ICB-induced increase of the IL-12/IL-10 ratio in λ-MYC TIDCs (Fig. 2c). In untreated tumor-bearing animals, this parameter was significantly reduced compared with healthy WT mice. This could be assigned to the action of IL-10, which was primarily produced by the tumor cells [24]. The capability of ICB therapy of reversing the cytokine imbalance in TIDCs may be due to direct mAb binding to PD-1 and CTLA-4 expressed on the DC surface (Fig. 3) as well as to an indirect effect mediated by IFN-γ [19, 20]. In a model of ovarian cancer, TIDCs also expressed PD-1 molecules on their surface, which were recognized by anti-PD-1 mAbs [32].

Experiments in vitro could not exclude that direct interactions of ICB mAbs with TIDCs expressing CTLA-4 and PD-1 may have impact on the IL12/IL-10 ratio (Fig. 3). In the in vivo situation, however, the cytokine expression pattern seemed to be mainly determined by IFN-γ, because ablation of IFN-γ during ICB therapy blocked the ICB-induced increase of the IL-12/IL-10 ratio (Fig. 2c). IFN-γ but not direct mAb binding to DCs may therefore be necessary in the in vivo setting, which is in accordance with another investigation, where DCs were similarly stimulated to express IL-12 via IFN-γ [33]. In the latter study, IFN-γ was derived from anti-PD-1-activated CD8^+^ T cells and eventually gave rise to activation of effector T cells. Further studies conducted in mouse tumor models indicate an activation of CD4^+^ T cells, which again stimulated DCs to express IL-12 [34]. The improved T cell-stimulatory capacity of λ-MYC TIDCs detected in vitro (Fig. 4), which was also seen in an HPV16 tumor model after anti-PD1/anti-CTLA-4 treatment [35], may be due to the altered cytokine expression pattern, but other mechanisms cannot be precluded.

Recent studies demonstrated an antimetastatic effect of NK cells induced by ICB in combination with IL-12 treatment [36] and ameliorated NK-cell functions as a result of reactivating specific CD4^+^ T cells [37]. Hence, reinvigoration of an exhausted adaptive immune response can give rise to an improved innate response [37]. Our results suggest that an additional, inverse pathway may exist, which is initiated by IFN-γ produced by activated NK cells, for example (Fig. 5). The lymphoma model presented here may reflect the situation in human melanoma where NK cells controlled DCs and stimulated a T-cell response through an NK cell/DC axis [38]. In summary, ICB therapy not only reactivates exhausted T cells but may also promote additional pathways, in which IFN-γ and DCs play a significant role.

## Electronic supplementary material

Below is the link to the electronic supplementary material.

Supplementary file1 (PDF 218 kb)
