# Supplementary material for: The role of dendritic cells for therapy of B-cell lymphoma with immune checkpoint inhibitors
Source: Cancer Immunol Immunother. 2020 Nov 3;70(5):1343–50. doi: 10.1007/s00262-020-02767-6 (PMC8053142; doi:10.1007/s00262-020-02767-6)
Supplement: Supplementary file 1 — Supplementary file1 (PDF 218 kb) [file 262_2020_2767_MOESM1_ESM.pdf]

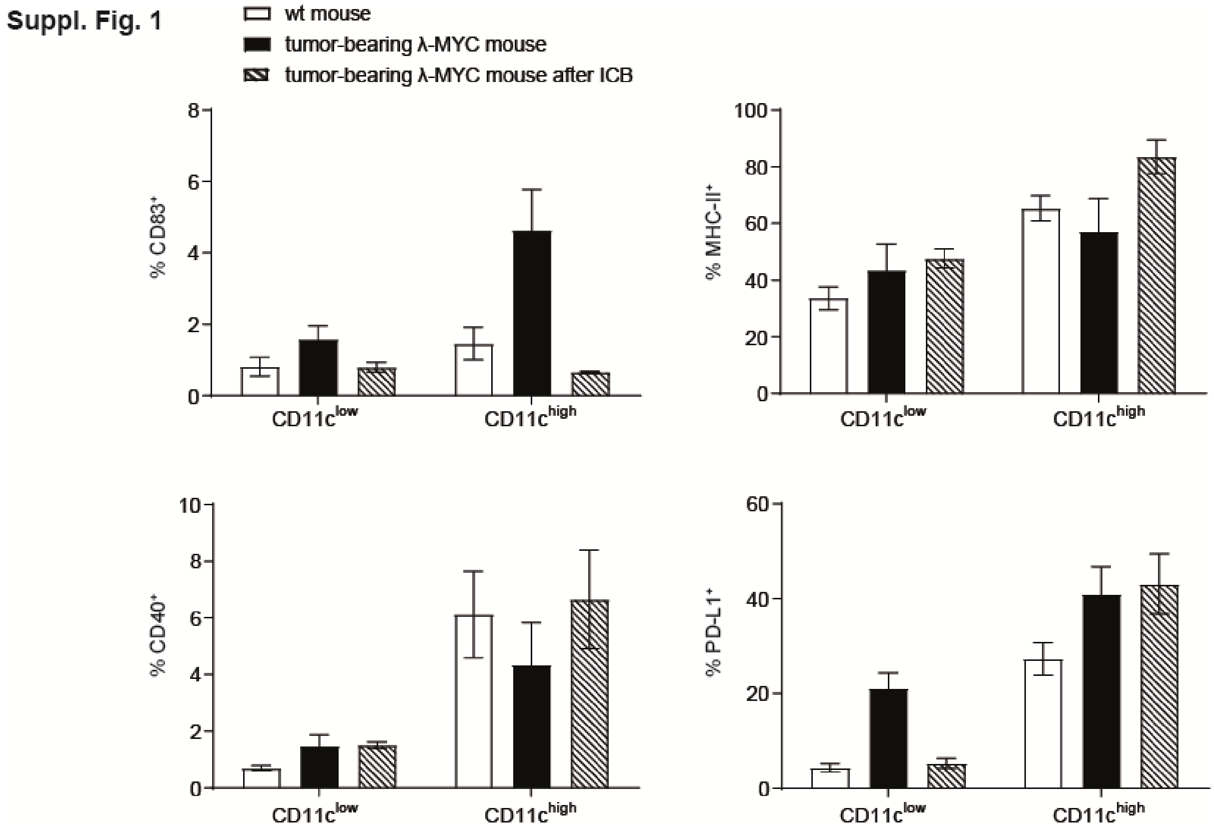

Supplementary Figure 1 Quantitation of CD83, CD40, MHCII and PD-L1 expressed on $CD11c^{low}$ and $CD11c^{high}$ spleen DCs from tumor-bearing λ-MYC mice that did or did not receive ICB treatment. WT mice are included for comparison. 4 to 10 animals were analyzed in the ICB and in the control group, respectively. A statistically significant effect of ICB therapy in comparison to the untreated group was only seen for CD83 in the $CD11c^{high}$ fraction and for PD-L1 in the $CD11c^{low}$ population ($P<0.05$ and $P<0.005$, respectively).
